# Frequency of the odontogenic maxillary sinusitis extended to the anterior ethmoid sinus and response to surgical treatment

**DOI:** 10.4317/medoral.19629

**Published:** 2014-03-08

**Authors:** Rafael Crovetto-Martínez, Francisco J. Martin-Arregui, Aitor Zabala-López-de-Maturana, Kiara Tudela-Cabello, Miguel A. Crovetto-de la Torre

**Affiliations:** 1 Department of Stomatology II. School of Medicine and Odontology, University of the Basque Country (UPV/EHU). Leioa. Vizcaya, Spain; 2 Department of Otorhinolaryngology. Basurto University Hospital (UPV/EHU), Bilbao, Vizcaya, Spain

## Abstract

Objectives: Odontogenic sinusitis usually affects the maxillary sinus but may extend to the anterior ethmoid sinuses. The purpose of this study is to determine the percentage of odontogenic maxillary sinusitis extended to the anterior ethmoid sinuses and determine also the surgical resolution differences between odontogenic maxillary sinusitis and odontogenic maxillary associated to anterior ethmoidal sinusitis. Study Design: This is a retrospective cohort study performed on 55 patients diagnosed of odontogenic sinusitis and treated surgically by functional endoscopic sinus surgery. Results: This study showed that 52.7% of odontogenic maxillary sinusitis spreads to anterior ethmoid, causing added anterior ethmoid sinusitis. We found that 92.3% of the odontogenic maxillary sinusitis (who underwent middle meatal antrostomy) and 96.5% of the odontogenic maxillary sinusitis extended to the anterior ethmoid (treated with middle meatal antrostomy and anterior ethmoidectomy) were cured. Conclusions: Ethmoid involvement is frequent in maxillary odontogenic sinusitis. The ethmoid involvement does not worsen the results of “functional endoscopic sinus surgery” applied to the odontogenic sinusitis.

** Key words:**Odontogenic maxillary sinusitis, ethmoiditis, functional endoscopic sinus surgery.

## Introduction

Maxillary sinusitis can be rhinogenous or odontogenic (OS), according to their origin, nasal or dental. OS differs from the rhinogenous sinusitis in its pathophysiology, microbiology and treatment (1). OS represents 10% to 40% of all maxillary sinusitis (2,3), and its incidence may be increasing (4,5).

Most of the OS are the result of periapical abscesses caused by caries or periodontal disease (6-8). Other causes are maxillary dental trauma, antral foreign bodies secondary to dental procedures (dental roots in traumatic extraction, dental materials, parts of broken instruments), oroantral fistula, placement of dental implants, sinus floor elevation procedures (1,6,9).

**Figure 1:**
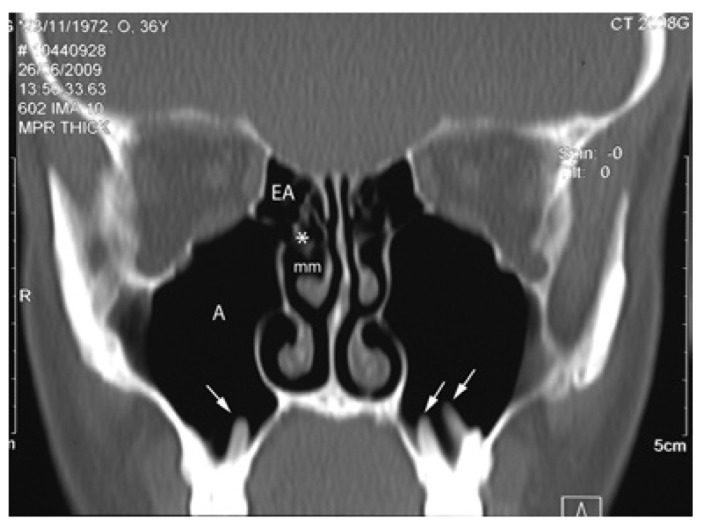
CT scan (coronal plane) of the maxillary and anterior ethmoid area corresponding to a healthy adult. The drainage ostium of the maxillary sinus (A) and those of the anterior ethmoid sinuses (EA), are located in close vicinity (*) in the middle meatus (mm). This image shows a close relationship of the dental roots with the maxillary sinus, which projecting into de antrum (arrows).

The anatomical proximity between the maxillary sinus ostium (natural drainage hole) and those of the anterior ethmoid sinus (Fig. 1) facilitate the spread of inflammation from the maxillary sinus to the anterior ethmoid cells, causing anterior ethmoiditis (10,11).

Computerized Tomography (CT) is considered to be the gold standard for OS (8,12,13). Initially odontogenic sinusitis is treated with antibiotics and decongestants, while the dentist performs a local treatment of dental disease, if possible. However, if the OS does not resolve with these measures, the treatment of choice is surgical drainage of the maxillary sinus (11). The surgical techniques used in the OS are the Caldwell-Luc approach or a “Functional Endoscopic Sinus Surgery” (FESS) (11,14).

The objective of this study is to establish the percentage of odontogenic maxillary sinusitis that extend to anterior ethmoid, causing anterior ethmoidal sinusitis, and determine differences in the outcome of surgical treatment (FESS) in odontogenic maxillary sinusitis with or without an added anterior ethmoidal sinusitis.

## Material and Methods

This is a retrospective cohort study. This study was conducted in the Department of Otolaryngology of Basurto University Hospital, with the collaboration of the Department of Stomatology II of the UPV / EHU. The Ethics Committee of Basurto Hospital approved this study.

The study included all patients who have undergone surgery for OS between January 2008 and December 2012, regardless of sex or age. No exclusion criteria were applied. The Ethics Committee of Basurto Hospital approved the study.

**Figure 2:**
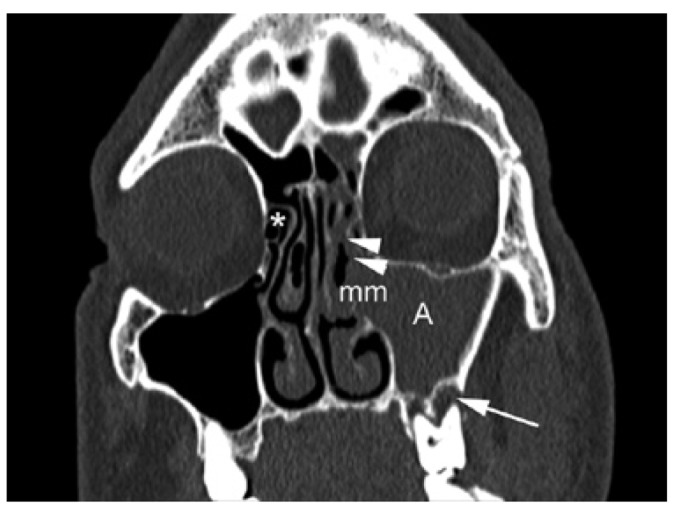
CT scan (coronal plane) corresponding to a 49 years old man patient showing a left odontogenic maxillary sinusitis extended to anterior ethmoid sinus. There are a diffuse opacification of the maxillary sinus (A) and anterior ethmoid sinus (arrowheads). The middle meatus (mm), where are situated the ostium of maxillary and anterior ethmoid sinus, is compromised by the inflammatory process. The anterior ethmoid sinuses on the right side are normal (*). The arrow indicates the location of a periapical lesion (abscess) adjacent to the sinus floor.

In all our patients, the suspected diagnosis of OS is based on clinical criteria and nasal and oral examinations, but multiplanar views CT make definitive diagnosis (Fig. 2). The radiological confirmation of odontogenic maxillary sinusitis has been made if total or partial opacification (>1/3 of the lumen) of the maxillary sinus affecting at least the antral floor. In addition, it should be added any of the following CT findings:

• Oroantral fistula.

• Periapical lesion defined as a rounded lucency adjacent the roots of a tooth in continuity with the maxillary sinus.

• Projecting tooth root above the floor of the antrum with a halo of lucency indicating periodontal disease.

• Dental roots, dental materials, implants, material for sinus augmentation, or parts of broken instruments in the maxillary sinus.

The diagnosis of anterior ethmoid sinusitis has been made with CT scan, when observing the anterior ethmoid occupation in continuity with antral involvement (Fig. 2). The average time between the onset of symptoms suggestive of OS and the realization of CT was superior in all cases at 6 months.

All patients accepted for surgical treatment have been refractory to medical treatment (combination of antibiotics and decongestants). The dentist has also treated them for dental disease, except in the case of oro-antral fistula or presence of teeth or foreign bodies in the maxillary sinus, in which case we have indicated a surgical solution.

Patients accepted for surgical treatment have been operated by FESS. Middle meatal antrostomy, consisting in an enlargement of the natural opening of the maxillary sinus into the middle meatus, was performed in the odontogenic maxillary sinusitis. In patients with odontogenic maxillary sinusitis extended to anterior ethmoidal sinus we performed middle meatal antrostomy and anterior ethmoidectomy, including at least the opening of the ethmoid bulla. Septoplasty or turbinoplasty was performed if access to the middle meatus was limited by septal deviation or thickening of the middle turbinate.

FEES were performed under general anaesthesia. Packing soaked in topical anesthetic solution with epinephrine decongests nasal mucosa, especially in middle meatus. Under control of a rigid 4 mm endoscope of 0º the middle turbinate was dislocated and uncinate process was trimmed to permit identification of the maxillary sinus ostium. The ostium is enlarged in a posterior and inferior route to complete an antrostomy greater than 140 mm2. Anterior ethmoidectomy started by opening the ethmoid bulla, completing ethmoidectomy depending on their degree of involvement. Rigid 4 mm endoscopes of 30º, 45º and 70º and curved suction, microdebrider and forceps was used to removal pathologic content and polyps, while swollen mucosa was unharmed, following the principles of the FESS (15,16). After surgery we perform nasal packing for 2 days. Patients remain hospitalized less than 24 hours and receive antibiotic treatment for 8 days. Three months after surgery were performed nasal endoscopic and radiological controls (CT), prior informed consent of the patients of the risks-benefits of this second scan radiation.

When sinusitis was associated with oroantral fistula we performed in the same surgical procedure a FESS approach and additional oral surgical treatment closing the fistulous defect with mucoperiosteal flap.

**Figure 3:**
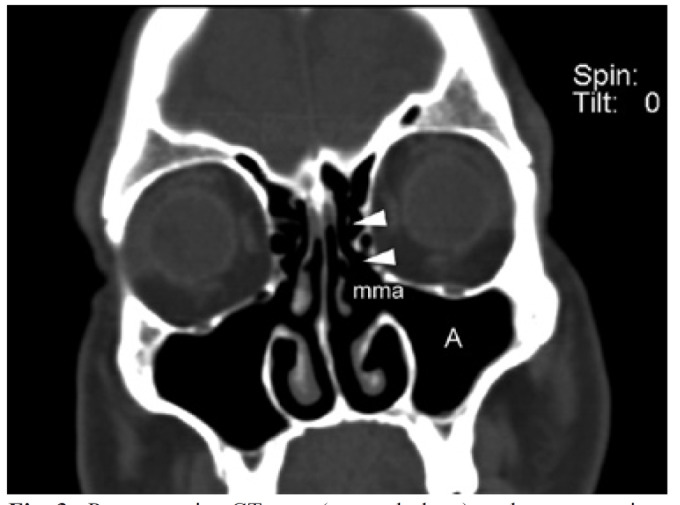
Postoperative CT scan (coronal plane) to the same patient of figure 2. The maxillary sinus (A) and the anterior ethmoid (arrowheads) are perfectly ventilated trough the middle meatal antrostomy (mma) associated to anterior ethmoidectomy, made by functional endoscopy sinus surgery.

Definition of cure: Three months after surgery the patient was asymptomatic, without signs of inflammation in middle meatus under nasal endoscopy and CT shows good ventilation in the maxillary and ethmoid sinuses, without effusion or thickening (>1/4 of the antral lumen) of the mucosa (Fig. 3). This is a precise method to evaluate the operated patients, as confirm other authors (17).

Definition of failure: Three months after surgery did not fulfil the necessary conditions for success.

Statistical Analysis: We have used descriptive statistics including frequency tables, means and standard deviations.

## Results

The study includes 55 patients operated on odontogenic sinusitis by FESS. Most of cases, 38 patients, presented periapical infection extending to the maxillary sinus floor (in 19 cases with a concomitant periodontal disease), 9 patients presented chronic oroantral fistula after molar extraction (7 cases) or premolar extraction (1 case) or implant extraction (1 patient), 1 patient had maxillary sinus infection secondary to peri-implantitis, 7 patients presented iatrogenic factors: placement of dental implant (1 case), intra-antral foreign bodies (4 cases) and sinus floor elevation and grafting procedures (2 patients). The mean age of the sample is of 47.8 (SD: 14.85). There are 33 males (60%) and 22 females.

94.5% of all OS surgically operated were cured ( Table 1). We performed two types of surgical approach, depending on the pathology found: middle meatal antrostomy made in odontogenic maxillary sinusitis (47.3%), and middle meatal antrostomy associated to anterior ethmoidectomy made in maxillary sinusitis extended to anterior ethmoid sinus (52.7%). We found that 92.3% of the odontogenic maxillary sinusitis and 96.5% of the odontogenic maxillary sinusitis associated to anterior ethmoid sinusitis were cured. We have not found relationship between different odontogenic sinusitis causes with success or failure of FESS ( Table 1).

**Table 1:**
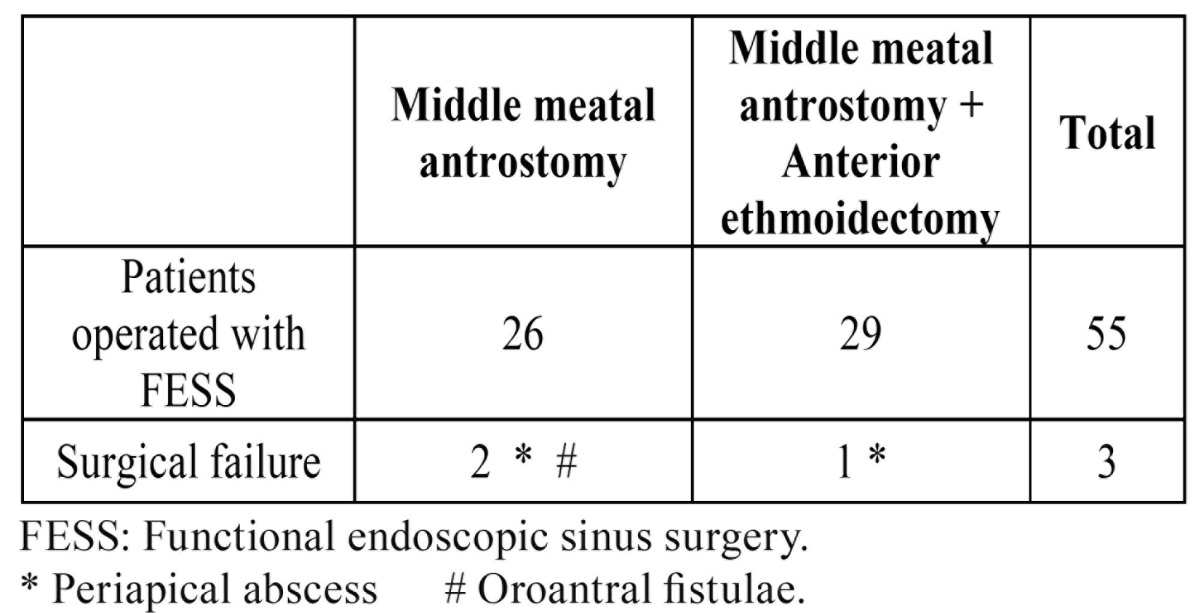
This table shows in right column the total number of patients with odontogenic sinusitis and surgical failures obtained after the first functional endoscopic sinus surgery (FESS). In the two central columns are exposed the subjects operated with the FESS surgical variants, and failures of each one of them.

In three, of 55 patients, failed the first surgery performed (2 of them underwent middle meatal antrostomy and 1 with associated anterior ethmoidectomy) due to postoperative synechiae in the middle meatus ( Table 1). None of these three patients, in whom the first FESS failure, had risk factors (local or general). The three underwent a new FESS, to eliminate synechiae and re-open the antrostomy, with healing result. There have been no notable complications in the 55 cases operated.

## Discussion

The mean patient age at the time of surgery was found to be 47,8, which is similar to that found by other authors in similar studies, being the fourth decade the most frequently affected by OS (18).

Concerning the analysis of patient gender with OS, previous works have found a similar prevalence or a slight female preponderance (57.7% in women) (13,18), but in our sample there is a discreet male preponderance (33 cases out of 55), which means that 60% of the sample were men.

Previous studies have identified an ethmoid involvement during OS, without determining its prevalence (10,19). We found that half of OS have maxillary and anterior ethmoid sinusitis. In these cases, the anterior ethmoiditis is probably the result of proximity of the maxillary sinus ostium and those of anterior ethmoid sinuses, which facilitate ethmoid inflammation from an infected maxillary sinus (20). Presumably, another crucial factor is related to the time factor, being that the longer evolutionary presents a maxillary sinusitis, more likely is that inflammation spreading to anterior ethmoid sinus causing an anterior ethmoid sinusitis. In this respect, it should be noted that the Spanish National Health System, where this study has been made, is free public, and that fact determines that the workload is very important and the waiting lists for hospital care are prolonged (weeks or months). In our study, the average time between the onset of the odontogenic sinusitis and performing CT has probably been more than six months, but this measurement may be inaccurate given the latent clinic of many OS.

Since the OS is not exclusively maxillary in more than half of the cases, their surgical approach by Caldwell-Luc technique that only addresses the maxillary sinus is, in our opinion, insufficient. The FESS, however, allows access to all inflamed sinuses: maxillary sinus and anterior ethmoid sinus. It could be argued that either of these two surgical approaches solves the maxillary sinusitis, whose recovery depends on the surgical antrostomy done by either technique. In fact, the antrostomy restore the ventilation and drainage of the maxillary sinus by ending vicious circle which self-perpetuating the sinusitis, due to the antral confinement. However, although the Caldwell-Luc technique achieves this purpose, must rely on spontaneous healing of the anterior ethmoiditis, but this is not necessary when using FESS, because the anterior ethmoiditis solves surgically (10).

On the other hand, the maxillary sinus has an effective mucociliary clearance leading the sinus secretions to the ostium of the maxillary sinus, to ensuring their effective drainage. This mucociliar function remains unchanged after sinus surgery. Middle meatal antrostomy facilitates mucociliary clearance by extending the maxillary ostium of the sinus. However, this does not happen in Caldwell-Luc because their artificial antrostomy is made in the inferior meatus, away from the natural ostium drainage (21,22).

Furthermore, the Caldwell-Luc surgical approach is associated with more complications than the FESS (23). In medium term, the Caldwell-Luc leaves an maxillary anterior bony wall defect, sinusal sclerosis and collapse of the sinus cavity (17). In our 55 patients undergoing FESS has not been any notable complication. Regarding clinical efficacy, FESS is equally or more effective than Caldwell-Luc to solve surgically OS, with less morbility (10,17). Lopatin *et al*. (14) were the first who reported 70 cases of odontogenic sinusitis treated by endoscopic sinus surgery (14), and since then the FESS is the surgical technique indicated in the treatment of OS (maxillary and maxillo-ethmoidal).

In our case, the global cure of OS by FESS is 94.5%. When we performed an isolated middle meatal antrostomy the 92.3% of maxillary sinusitis have healed, while the percentage of healing after middle meatal antrostomy and anterior ethmoidectomy, because a maxillary and anterior ethmoid sinusitis, was 96.5%. These results allow us to infer that the ethmoid involvement does not worsen the prognosis of the odontogenic sinusitis treated with nasal endoscopic surgical approach (FESS).

## Conclusions

Half of the patients with an odontogenic maxillary sinusitis also present an anterior ethmoiditis. The healing of odontogenic sinusitis by endoscopic sinus surgery (FESS) is achieved in 94.5% after the first intervention, reaching a 100% cure after re-intervention. Ethmoid involvement does not worsen the FESS surgical results applied to the odontogenic sinusitis.
